# A chemically self-charging aqueous zinc-ion battery

**DOI:** 10.1038/s41467-020-16039-5

**Published:** 2020-05-04

**Authors:** Yan Zhang, Fang Wan, Shuo Huang, Shuai Wang, Zhiqiang Niu, Jun Chen

**Affiliations:** Key Laboratory of Advanced Energy Materials Chemistry (Ministry of Education), Renewable Energy Conversion and Storage Center, College of Chemistry, Nankai University, Tianjin, 300071 People’s Republic of China

**Keywords:** Batteries, Batteries

## Abstract

Self-charging power systems integrating energy harvesting technologies and batteries are attracting extensive attention in energy technologies. However, the conventional integrated systems are highly dependent on the availability of the energy sources and generally possess complicated configuration. Herein, we develop chemically self-charging aqueous zinc-ion batteries with a simplified two-electrode configuration based on CaV_6_O_16_·3H_2_O electrode. Such system possesses the capability of energy harvesting, conversion and storage simultaneously. It can be chemically self-recharged by the spontaneous redox reaction between the discharged cathode and oxygen from the ambient environment. Chemically self-recharged zinc-ion batteries display an initial open-circuit voltage of about 1.05 V and a considerable discharge capacity of about 239 mAh g^−1^, indicating the excellent self-rechargeability. Impressively, such chemically self-charging zinc-ion batteries can also work well at chemical or/and galvanostatic charging hybrid modes. This work not only provides a route to design chemically self-charging energy storage, but also broadens the horizons of aqueous zinc-ion batteries.

## Abstract (web summary)

Self-charging power systems integrating energy generation and storage are receiving consideration attention. Here the authors report an aqueous Zn-ion battery that can be self-recharged by the spontaneous redox reaction between cathode and oxygen from ambient environment without external power supply.

## Introduction

Rechargeable batteries are widely used in many fields, such as electric devices and grid-scale energy storage systems^1–4^. In general, the commercial batteries are often charged by electrical grid. However, in the harsh environment or remote area, the electrical grid is unavailable, which limits the recharging and reuse of batteries. In order to solve this issue, various energy harvesting technologies, such as photovoltaic devices^5–11^, piezoelectric nanogenerators^12,13^, triboelectric nanogenerators^14,15^, and thermoelectrics^16–18^, were integrated with batteries into self-charging power systems. However, these systems are highly dependent on the energy resources, which are not always available in some environment or during a certain period of time. Furthermore, the configurations of these integrated systems are usually complicated and many extra components (e.g., photoelectrodes or temperature-sensitive redox couples) are required in comparison with the traditional batteries with two-electrode configuration. Therefore, self-charging power systems that possess simplified configuration and are available in various environments must be considered.

Chemical energy stored in molecules is an available energy source and can be converted into electrical energy through redox reaction^19–22^. In this regard, chemical energy of oxygen that is an abundant resource in air is attracting much more attention in the energy conversion and storage devices, such as metal–air batteries. Furthermore, the metal–air batteries can be further integrated with other energy storage devices and charge them^23,24^. However, in such integrated devices, the metal–air components cannot always compensate the energy consumption of energy storage devices, and they have to be recharged by external power supply to recover when both the metal–air and energy storage components are exhausted^23^. Therefore, they cannot meet the demand in some case, where the batteries are required to be charged directly by the successive chemical energy conversion of oxygen on their cathodes. Recently, a variety of vanadium-based compounds have been developed to serve as the cathode materials of aqueous zinc-ion batteries (ZIBs) due to their open-framework crystal structure and multiple oxidation states of vanadium^25–30^. During the discharge process, the insertion of Zn^2+^ ions and reduction of vanadium occur simultaneously in vanadium-based ZIBs. Furthermore, it is noted that vanadium-based compounds are also active redox materials and can be oxidized by oxygen in low valence state. Therefore, the discharged vanadium-based cathodes would be oxidized by oxygen in the ambient environment, like a charge process. As a result, self-charging ZIBs that simultaneously possess energy conversion and storage functions would be achieved.

Inspired by this, we develop a chemically self-charging aqueous ZIBs system, in which the chemical energy harvesting, conversion, and storage are integrated in a single CaV_6_O_16_·3H_2_O (CaVO) cathode. Such system possesses a similar two-electrode configuration with the conventional ZIBs. It can harvest energy from ambient environment through spontaneous redox reaction, and then convert chemical energy into electrical energy and store them in ZIBs. Therefore, the resultant ZIBs can be self recharged by directly exposing CaVO cathodes to air without any external power supply. This design would provide a promising research direction for the next-generation self-powered systems.

## Results

### Preparation and characterization of CaVO nanoribbons

**Fig. 1:**
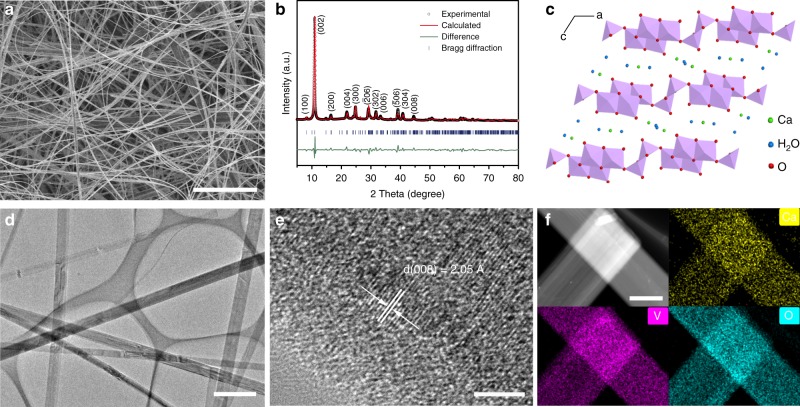
Morphology and crystal structure of CaVO nanoribbons. **a** SEM image. **b** Rietveld refinement of the XRD pattern. **c** Crystal structure. **d** TEM image. **e** HRTEM image. **f** TEM elemental mapping images. Scale bars: **a** 10 μm; **d** 1 μm; **e** 3 nm; and **f** 400 nm.

The CaVO nanoribbons were synthesized via a one-step hydrothermal method^31^. They display a size of several hundred micrometers in length and 200–500 nm in width (Fig. 1a, d). Their crystalline phase was further understood by X-ray diffraction (XRD) and the corresponding Rietveld refinement, as shown in Fig. 1b. The results show that the diffraction peaks can be well indexed to the monoclinic CaVO phase with space group of A2/m (JCPDS 33-317) and lattice parameters of *a* = 12.2533 Å, *b* = 3.5149 Å, *c* = 18.3874 Å, *α* = *γ* = 90.0°, and *β* = 118.642°. Thermogravimetric analysis (TGA) also confirms that three molecular waters exist in a formula unit (Supplementary Fig. 1, Supplementary Note 1). Furthermore, it is noted that the XRD pattern exhibits multiple (00*l*) reflections and the intensity of (002) reflection is extremely strong. It suggests that CaVO nanoribbons possess a typical layered structure, which is constructed by the stacking of V_6_O_16_ framework and the hydrated Ca atoms act as “pillars” between the V_6_O_16_ layers to stabilize the layered structure (Fig. 1c)^32,33^. In addition, the lattice fringes with *d*-spacing of 0.205 nm is observed in the high-resolution transmission electron microscopy (HRTEM) image, corresponding to the (008) plane of CaVO (Fig. 1e), which is in good agreement with the XRD result. Moreover, the transmission electron microscopy (TEM) elemental mapping images clearly show that Ca, V, and O element are homogeneously distributed in CaVO nanoribbons (Fig. 1f).

### Electrochemical performance of Zn/CaVO batteries

**Fig. 2:**
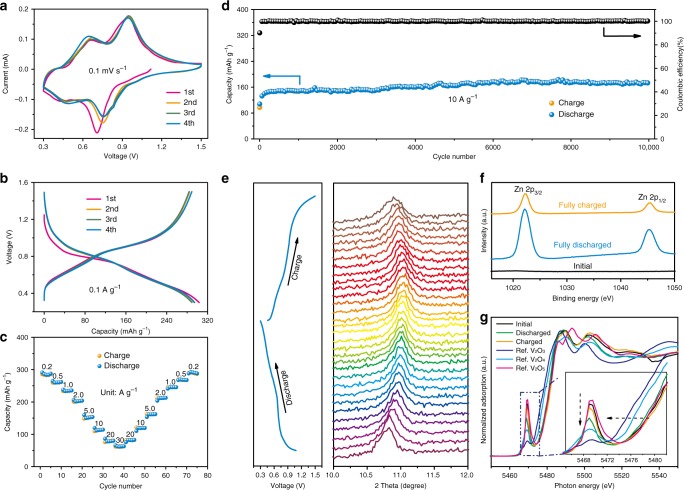
Electrochemical performance and mechanism of Zn/CaVO batteries. **a** CV curves at 0.1 mV s^−1^. **b** GCD curves at 0.1 A g^−1^. **c** Rate capability at various current densities. **d** Cycling performance at 10 A g^−1^. **e** In situ XRD patterns of (002) reflection and corresponding GCD curves at 0.2 A g^−1^ during the first cycle. **f** XPS spectra of Zn 2p at initial, fully discharged, and charged states. **g** V K-edge XANES curves at initial, fully discharged, and charged states with reference to the standard V_2_O_3_, VO_2_, and V_2_O_5_.

The favorable morphological features, open framework and expanded interlayer spacing (0.819 nm) would contribute to the fast kinetics of Zn^2+^ ions insertion/extraction in CaVO (refs. ^26,34–36^), thereby enhancing their electrochemical performance. The electrochemical performance of CaVO nanoribbons was investigated in coin cells. In their cyclic voltammetry (CV) curves, there are two pairs of reduction/oxidation peaks at 0.75/0.95 and 0.47/0.66 V (Fig. 2a). They can be ascribed to a two-step redox reaction associated with Zn^2+^ ion insertion/extraction (corresponding analysis see the following energy storage mechanism section), corresponding to the valence change of vanadium from V^5+^ to V^4+^ and V^4+^ to V^3+^, respectively^26,37,38^. Furthermore, the CV curves display similar shape after first cycle, suggesting the high reversibility of the charge/discharge process of Zn/CaVO batteries, which is further confirmed by the reversible galvanostatic charge/discharge (GCD) profiles (Fig. 2b). Besides, Zn/CaVO batteries deliver a high initial discharge capacity of 300 mA h g^−1^ at 0.1 A g^−1^ and an average operating voltage of ~0.7 V versus Zn^2+^/Zn, corresponding to an insertion of ~3.6 Zn per unit formula of CaVO. Impressively, even the current density is up to 30 A g^−1^, a capacity of 62 mAh g^−1^ is still achieved (Fig. 2c). When the current density recovers from 30 to 0.2 A g^−1^ after 70 cycles, the capacity is able to recover to the initial capacity of ~290 mAh g^−1^. Such excellent rate capability of Zn/CaVO batteries significantly depends on their electrochemical kinetics, as suggested by CV measurements at different scan rates (see Supplementary Fig. 2 and detailed calculation in Supplementary Note 2). The high surface-controlled capacity contributions result in the fast Zn^2+^ ion diffusion, enabling the high rate capability as well as outstanding cycling stability^39–42^. The capacity of Zn/CaVO batteries is still 263 mAh g^−1^ after 100 cycles at 0.5 A g^−1^ (Supplementary Fig. 3). Impressively, even after 10,000 cycles at a high current density of 10 A g^−1^, there is no degradation in their capacity, remaining stable at ~170 mA h g^−1^ (Fig. 2d). During initial 200 cycles, the capacity is gradually increased, which is attributed to the activation of CaVO at a high current density of 10 A g^−1^ (refs. ^43,44^).

### Energy storage mechanism of Zn/CaVO batteries

The fast reaction kinetics and excellent electrochemical performance depend on the energy storage mechanism of Zn/CaVO batteries. To further understand the energy storage mechanism of Zn/CaVO system, various measurements, including in situ/ex situ XRD, ex situ TEM, X-ray photoelectron spectrometry (XPS) and X-ray absorption near-edge spectroscopy (XANES) were utilized to characterize the CaVO cathodes at the selected states of charge/discharge process. It is noted that the XRD characteristic diffraction peaks of (002) and (004) plane shift slightly toward high degree during the discharge process, corresponding to the decrease of interlayer spacing (Fig. 2e, Supplementary Figs. 4 and 5). It is ascribed to the strong electrostatic interaction between intercalated Zn^2+^ ions and V_6_O_16_ layers^38,45^. In contrast, the ($\bar{2}06$) reflection shifts slightly toward low degree, corresponding to the expansion of the interlayer spacing of ($\bar{2}06$) plane, which could arise from the reduction of V^5+^/V^4+^ upon Zn^2+^ ion insertion and the resultant increase in the V–V bond distance between the layers^43^. Furthermore, the disorder in structure caused by Zn^2+^ ion insertion would result in the broadening of ($\bar{2}06$) and (006) reflections. Reversibly, during the subsequent charge process, these characteristic diffraction peaks gradually return to their original state with the extraction of Zn^2+^ ions. This reversible change of interlayer spacing is also confirmed by the HRTEM images (Supplementary Fig. 6). Therefore, the energy storage mechanism of Zn/CaVO batteries is the insertion/extraction of Zn^2+^ ions into/from the CaVO (Supplementary Fig. 7, Supplementary Note 3), which is similar to the case of conventional ZIBs (refs. ^30,46^). Moreover, this Zn^2+^ ion insertion/extraction mechanism is further substantiated by the XPS analysis of Zn 2p peaks (Fig. 2f). The peaks of Zn 2p are not detected in the initial CaVO cathode, whereas strong peaks are observed at the fully discharged state, corresponding to the intercalated Zn^2+^ ions. In contrast, at the fully charged state, the intensity of Zn 2p peaks is significantly weakened, suggesting that most of Zn^2+^ ions are extracted from the CaVO. It is also proved by TEM elemental mapping images, where Zn element is uniformly distributed in the nanoribbons at fully discharged state and then almost disappears at fully charged state (Supplementary Fig. 8). The insertion/extraction of Zn^2+^ ions is accompanied by the valence change of vanadium in CaVO, as illustrated in V K-edge XANES curves of CaVO cathodes at different charge/discharge states (Fig. 2g). Compared with the initial state, the main absorption edge slightly shifts toward lower binding energy at fully discharged state, suggesting that the average oxidation state of vanadium is reduced due to the insertion of Zn^2+^ ions^47–49^. Subsequently, the position of absorption edge reversibly shifts back toward higher energy at fully charged state, indicating the oxidation of vanadium caused by the extraction of Zn^2+^ ions from CaVO. The above insertion/extraction of Zn^2+^ ions during the charge/discharge process can also be reflected by the reversible intensity change of pre-edge peak in XANES (Fig. 2g), which is sensitive to the change of the local symmetry of V atoms^50^.

Based on the above results and discussion, the overall electrochemical reaction of the Zn/CaVO batteries can be described as follows:

Cathode:

1 $${\mathrm{CaV}}_6{\mathrm{O}}_{16} \cdot 3{\mathrm{H}}_2{\mathrm{O}} + 3.6{\mathrm{Zn}}^{2 +} + 7.2e^ - \leftrightarrow {\mathrm{CaZn}}_{3.6}{\mathrm{V}}_6{\mathrm{O}}_{16} \cdot 3{\mathrm{H}}_2{\mathrm{O}}$$

Anode:

2 $$3.6{\mathrm{Zn}} \leftrightarrow 3.6{\mathrm{Zn}}^{2 + } + 7.2e^ -$$

Overall:

3 $${\mathrm{CaV}}_6{\mathrm{O}}_{16} \cdot 3{\mathrm{H}}_2{\mathrm{O}} + 3.6{\mathrm{Zn}} \leftrightarrow {\mathrm{CaZn}}_{3.6}{\mathrm{V}}_6{\mathrm{O}}_{16} \cdot 3{\mathrm{H}}_2{\mathrm{O}}$$

### Chemically self-charging mechanism

**Fig. 3:**
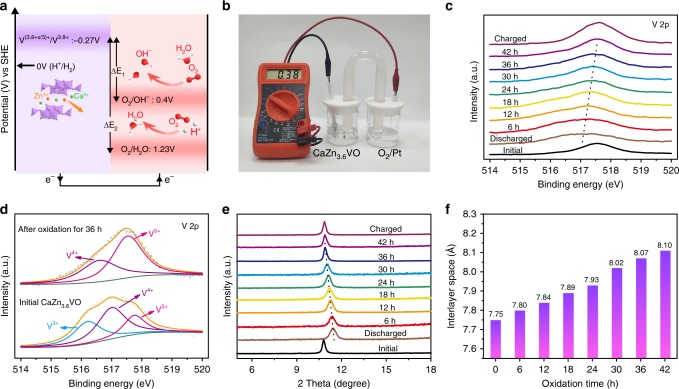
The mechanism of the redox reaction between CaZn_3.6_VO and O_2_. **a** Energy level transition diagram of CaZn_3.6_VO and O_2_. **b** Optical image of the designed galvanic cell. **c** V 2p XPS spectra of the CaVO electrodes at different states and the CaZn_3.6_VO electrodes after being oxidized by O_2_ in 4 M Zn (CF_3_SO_3_)_2_ solution for different times. **d** Comparison of XPS spectra of V 2p before and after oxidation. **e** XRD patterns and **f** calculational interlayer spacing of (002) plane of CaZn_3.6_VO electrodes after being oxidized by O_2_ for different times.

The CaVO cathodes exhibit excellent Zn^2+^ ion storage performance, and the reduction/oxidation of vanadium occurs during the Zn^2+^ ion insertion/extraction. In the galvanostatic charging process, electrons are released from the CaZn_3.6_V_6_O_16_·3H_2_O (CaZn_3.6_VO) cathode. As a result, vanadium in CaZn_3.6_VO is oxidized and the Zn^2+^ ions are extracted from the layered structure simultaneously. In this process, the driving force of the release of electron from CaZn_3.6_VO is generally the external power supply. In addition to above-mentioned electrochemical oxidation reaction, other strategies that can realize the electron transfer from CaZn_3.6_VO would be also promising to carry out the charging process of the ZIBs based on CaZn_3.6_VO cathode. It is well known that redox reaction is an effective and straightforward approach to realize the electron transfer, which is driven by the redox potential difference (Δ*E*) between reactants^51,52^. In the case of CaZn_3.6_VO, the redox potential in this system can be obtained from the CV curves of Zn/CaVO batteries (Fig. 2a)^53^, in which the minimum reduction peak potential of CaVO versus Zn^2+^/Zn is 0.47 V. According to the Nernst equation, the calculated redox potential of CaZn_3.6_VO is −0.27 V versus standard hydrogen electrode (SHE). Theoretically, CaZn_3.6_VO can be oxidized spontaneously by the oxidants with a higher potential than −0.27 V versus SHE. Among various oxidants, O_2_ is common and abundant in the air, the standard electrode potentials (*E*^*θ*^) of which are ~0.40 V and ~1.23 V versus SHE in the neutral and acidic medium, respectively. Owing to the difference in redox potential between O_2_ and CaZn_3.6_VO, the CaZn_3.6_VO tends to release electrons to be oxidized and O_2_ can accept these electrons to be reduced simultaneously (Fig. 3a). Therefore, if the redox reaction between CaZn_3.6_VO and O_2_ could take place, the oxidation of vanadium in CaZn_3.6_VO would be realized, and the Zn^2+^ ions would be extracted from the layered structure to balance the charge at the same time. As a result, the fully discharged product CaZn_3.6_VO would recover to its charged states CaZn_3.6−x_V_6_O_16_·3H_2_O (CaZn_3.6−x_VO) without any external power supply. In the Zn/CaVO batteries, acidic 4 M Zn(CF_3_SO_3_)_2_ electrolyte was used. In an open device, O_2_ could be dissolved into such electrolyte and imported to the discharged product CaZn_3.6_VO, realizing the redox reaction between CaZn_3.6_VO and O_2_. In order to validate the spontaneity of the redox reactions between CaZn_3.6_VO and O_2_ in the acidic 4 M Zn(CF_3_SO_3_)_2_ electrolyte, we designed a galvanic cell^54,55^

$$( - ){\mathrm{CaZn}}_{3.6}{\mathrm{VO}}|{\mathrm{CaZn}}_{3.6 - {\mathrm{x}}}{\mathrm{VO}}||{\mathrm{H}}_2{\mathrm{O}}|{\mathrm{O}}_2|{\mathrm{Pt}}( + )$$

As shown in Fig. 3b, the CaZn_3.6_VO electrode served as anode, which was in 4 M Zn(CF_3_SO_3_)_2_ electrolyte without the dissolved oxygen and sealed under Ar. The platinum wire was used as cathode, which was immersed in 4 M Zn(CF_3_SO_3_)_2_ solution containing dissolved oxygen. In this system, a cell voltage of 0.38 V was observed. According to the relationship between thermodynamic function and cell voltage:

4 $$\Delta G = - nEF$$

where, Δ*G*, *F*, and *E* are the free energy change, Faraday constant, and cell voltage, respectively, the free energy change is less than zero in this case, suggesting that the redox reaction between CaZn_3.6_VO and O_2_ can take place spontaneously in 4 M zinc trifluoromethanesulfonate (Zn (CF_3_SO_3_)_2_) solution at ambient condition.

To gain insight into the process of such redox reaction, various ex situ characterizations were carried out to investigate the structure and composition evolution of CaZn_3.6_VO electrodes that were immersed in 4 M Zn (CF_3_SO_3_)_2_ to react with the dissolved oxygen for different times. With the extension of immersion time from 0 (fully discharged state) to 36 h, the V 2p peak in the XPS spectra gradually shifts toward higher binding energy (Fig. 3c), where the intensity of V^5+^ peak is significantly enhanced and V^3+^ peak disappears in comparison with those of initial CaZn_3.6_VO (Fig. 3d), suggesting the oxidation of vanadium in CaZn_3.6_VO during the redox reaction process. Simultaneously, to balance the charge of CaZn_3.6_VO, the intercalated Zn^2+^ ions are extracted from the layered structure, resulting in the increase of the interlayer spacing of CaZn_3.6_VO, as proved by the XRD pattern. Furthermore, it is worth noting that the amount of extracted Zn^2+^ ions of CaZn_3.6_VO can be effectively controlled by varying the oxidation time in this system. The (002) reflection of oxidized CaZn_3.6_VO gradually shifts toward lower degree with the extension of oxidation time (Fig. 3e, Supplementary Figs. 5 and 9, Supplementary Note 4). According to the Bragg equation (2*dsinθ* = *nλ*, *n* = 1, *λ* = 0.154 nm), the interlayer spacing of (002) plane is calculated and increases from 7.75 to 8.10 Å after oxidation for 42 h (Fig. 3f). In addition, it is noted that V 2p XPS spectra and the (002) reflection will not further shifts when the oxidation time exceeds 36 h (Fig. 3c, e), suggesting the oxidation of vanadium in CaZn_3.6_VO and the extraction of Zn^2+^ ions would be finished within 36 h.

In addition to O_2_, it is noted that H_2_O also plays an important role in the redox reaction process. In order to demonstrate this, the CaZn_3.6_VO electrodes were immersed in acetonitrile containing dissolved oxygen. In the absence of H_2_O, the oxidation of vanadium is not detected in the resultant CaZn_3.6_VO, as reflected by the V 2p XPS spectra, where V 2p region remains unchanged in comparison with that of the initial CaZn_3.6_VO (Supplementary Fig. 10). Furthermore, there is no shift in the XRD characteristic peak of the resultant CaZn_3.6_VO (Supplementary Fig. 11), indicating that the Zn^2+^ ions are not extracted from the CaZn_3.6_VO. Clearly, the redox reaction between CaZn_3.6_VO and O_2_ cannot take place in the absence of H_2_O. In case of the neutral aqueous solution, the redox reaction between CaZn_3.6_VO and O_2_ can also occur (Supplementary Figs. 12–16, Supplementary Notes 5 and 6), where O_2_ accepts the electrons from CaZn_3.6_VO and reacts with H_2_O to form OH^−^. The generated OH^−^ and extracted Zn^2+^ ions combine with the adsorbed electrolyte ions (Zn^2+^ and CF_3_SO_3_^−^) to form amorphous triflate containing layered double hydroxide Zn_x+y_(CF_3_SO_3_)_2y_(OH)_2×_ (see Supplementary Figs. 17–22 and discussion in Supplementary Notes 7–10)^37,44,56,57^. Based on the above discussion, in the neutral deionized water, the redox reaction between CaZn_3.6_VO and O_2_ can be summarized as below:

5 $$\begin{array}{l}{\mathrm{CaZn}}_{3.6}{\mathrm{V}}_6{\mathrm{O}}_{16} \cdot 3{\mathrm{H}}_2{\mathrm{O}} + x/2{\mathrm{O}}_2 + x{\mathrm{H}}_2{\mathrm{O}} + y{\mathrm{Zn}}({\mathrm{CF}}_3{\mathrm{SO}}_3)_2 \to \\ {\mathrm{CaZn}}_{3.6 - {\mathrm{x}}}{\mathrm{V}}_6{\mathrm{O}}_{16} \cdot {\mathrm{3}}{\mathrm{H}}_2{\mathrm{O}} + {\mathrm{Zn}}_{{\mathrm{x}} + {\mathrm{y}}}({\mathrm{CF}}_3{\mathrm{SO}}_3)_{{\mathrm{2y}}}{\mathrm{(OH)}}_{2{\mathrm{x}}}\end{array}$$

Different from the case in neutral deionized water, in the acidic 4 M Zn(CF_3_SO_3_)_2_ electrolyte, O_2_ is reduced to H_2_O in the presence of H^+^ ions, and the CaZn_3.6_VO is oxidized along with the extraction of Zn^2+^ ions. The redox reaction mechanism can be expressed by the following half-cell reactions:

6 $${\mathrm{CaZn}}_{3.6}{\mathrm{V}}_6{\mathrm{O}}_{16} \cdot {\mathrm{3}}{\mathrm{H}}_2{\mathrm{O}} \to {\mathrm{CaZn}}_{3.6 - {\mathrm{x}}}{\mathrm{V}}_6{\mathrm{O}}_{16} \cdot 3{\mathrm{H}}_2{\mathrm{O}}\\ + x{\mathrm{Zn}}^{2 + } + 2xe^ -$$

7 $$x/2{\mathrm{O}}_2 + 2x{\mathrm{H}}^ + + 2xe^ - \to x{\mathrm{H}}_2{\mathrm{O}}$$

The continuous consumption of H^+^ ions promotes the water dissociation, thus producing additional H^+^ ions and OH^−^ ions:

8 $$2x{\mathrm{H}}_2{\mathrm{O}} \to 2x{\mathrm{H}}^ + + 2{\it{x}}{\mathrm{OH}}^ -$$

The additional H^+^ ions also participate in the oxygen reduction reaction (O_2_ + 4 H^+^ + 4e^−^ → 2H_2_O). The generated OH^−^ ions react with electrolyte ions (Zn^2+^ and CF_3_SO_3_^−^) and Zn^2+^ ions that are extracted from the oxidation reaction of CaZn_3.6_VO to form Zn_x+y_(CF_3_SO_3_)_2y_(OH)_2x_ on the surface of electrode (Supplementary Figs. 23 and 24):

9 $$x{\mathrm{Zn}}^{2 + } + y{\mathrm{Zn}}({\mathrm{CF}}_3{\mathrm{SO}}_3)_2 + 2x{\mathrm{OH}}^ - \to {\mathrm{Zn}}_{{\mathrm{x}} + {\mathrm{y}}}({\mathrm{CF}}_3{\mathrm{SO}}_3)_{2{\mathrm{y}}}({\mathrm{OH}})_{2{\mathrm{x}}}$$

The overall reaction can be expressed as:

$${\mathrm{CaZn}}_{3.6}{\mathrm{V}}_6{\mathrm{O}}_{16} \cdot 3{\mathrm{H}}_2{\mathrm{O}} + x/2{\mathrm{O}}_2 + x{\mathrm{H}}_2{\mathrm{O}} + y{\mathrm{Zn}}({\mathrm{CF}}_3{\mathrm{SO}}_3)_2 \to$$

10 $${\mathrm{CaZn}}_{3.6 - {\mathrm{x}}}{\mathrm{V}}_6{\mathrm{O}}_{16} \cdot 3{\mathrm{H}}_2{\mathrm{O}} + {\mathrm{Zn}}_{{\mathrm{x}} + {\mathrm{y}}}({\mathrm{CF}}_3{\mathrm{SO}}_3)_{2{\mathrm{y}}}{\mathrm{(OH)}}_{2{\mathrm{x}}}$$

### Self-charging performance of ZIBs

**Fig. 4:**
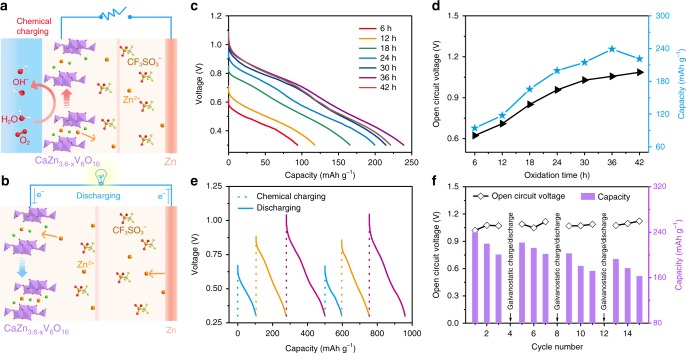
Chemical charging/galvanostatic discharging behavior of Zn/CaZn_3.6−x_VO batteries. Working mechanism of chemically self-charging ZIBs during **a** chemical charging and **b** galvanostatic discharging process. **c** The galvanostatic discharge curves of Zn/CaZn_3.6−x_VO batteries at 0.1 A g^−1^ after the CaZn_3.6_VO electrodes were oxidized for different times. **d** Effect of the oxidation time on OCV and discharge capacity of Zn/CaZn_3.6−x_VO batteries. **e** Voltage–time curves of the Zn/CaZn_3.6−x_VO batteries after being chemically charged to different states (dotted lines: chemical charging for different times. Solid lines: galvanostatic discharging at 0.1 A g^−1^). **f** Chemical charging/galvanostatic discharging cycling stability of the Zn/CaZn_3.6−x_VO battery in a voltage window from 0.3 to ~1.05 V.

The oxidation of vanadium and extraction of Zn^2+^ ions in CaZn_3.6_VO can spontaneously occur in the 4 M Zn(CF_3_SO_3_)_2_ electrolyte containing dissolved oxygen. Consequently, the fully discharged product CaZn_3.6_VO can recover to its charged states CaZn_3.6−x_VO through spontaneous redox reaction, which could be considered as a chemical self-charging process (Fig. 4a). When the oxidized product CaZn_3.6−x_VO electrode directly act as the cathode of ZIBs, the discharge process will take place due to the Zn^2+^ ion chemical potential difference between CaZn_3.6−x_VO cathode and Zn anode, where anodic Zn is dissolved to form Zn^2+^ ions and then intercalate into the CaZn_3.6−x_VO cathode (Fig. 4b)^51^. Simultaneously, electrons flow out of the anode to cathode through the external circuit to maintain electrical neutrality. In order to investigate the electrochemical performance of aqueous ZIBs based on CaZn_3.6−x_VO cathodes, their galvanostatic discharge curves were measured. The open-circuit voltage (OCV) of the Zn/CaZn_3.6−x_VO batteries rises continually along with extending the oxidation time of CaZn_3.6_VO (Fig. 4c, d). It is ascribed to the enhanced electrode potential of CaZn_3.6−x_VO cathode as a result of the deeper oxidation of vanadium and the extraction of more Zn^2+^ ions, with the extension of oxidation time. When the CaZn_3.6_VO is oxidized for 36 and 42 h, the OCV of Zn/CaZn_3.6−x_VO batteries reaches up to 1.05 and 1.08 V, respectively, which nearly reach to the initial OCV (~1.2 V) of the Zn/CaVO battery. In addition to OCV, the discharge capacity of Zn/CaZn_3.6-x_VO batteries also depends on the oxidation time of CaZn_3.6_VO. When the oxidation time is <36 h, the discharge capacity gradually increases with the prolongation of oxidation time and a discharge capacity of 239 mAh g^−1^ is achieved at 36 h. It is much higher than the case of chemical charging in neutral deionized water (Supplementary Figs. 25 and 26, Supplementary Note 11). However, it is noted that the discharge capacity of Zn/CaZn_3.6−x_VO batteries degrades when the oxidation time exceeds 36 h, since the increasing Zn_x+y_(CF_3_SO_3_)_2y_(OH)_2x_ would hinder the subsequent insertion of Zn^2+^ ions. In addition, Zn/CaZn_3.6−x_VO batteries display high reversibility of such chemical charging/galvanostatic discharging process even at different chemically self-charged states. When the chemical charging time is extended stepwise from 6 to 30 h and then abruptly switched back to 6 h, the corresponding discharge capacity increases from 105.1 to 221.4 mAh g^−1^ and then returns to 96.4 mAh g^−1^ (Fig. 4e). During the repeated chemical charging/galvanostatic discharging cycles, CaZn_3.6−x_VO can be reversibly converted with CaZn_3.6_VO (Supplementary Fig. 5). Different from CaZn_3.6−x_VO, the Zn_x+y_(CF_3_SO_3_)_2y_(OH)_2x_ is progressively generated and aggregated on the electrode, which will inevitably increase electrochemical impedance and hinder the insertion of Zn^2+^ ions during subsequent cycles, resulting in the capacity degradation (Fig. 4f). Importantly, after the electrochemical charging process, most Zn_x+y_(CF_3_SO_3_)_2y_(OH)_2x_ would be decomposed and disappear from the electrode (Supplementary Fig. 27, Supplementary Note 12). Therefore, the chemical-charging ability and capacity will recover to a large extent after the electrochemical charging process. However, it is noted that there is still a capacity decay even after several chemical-recharging/galvanostatic-recharging cycling (Fig. 4f). It could be ascribed to the structural stress generated from continuous extraction/insertion of Zn^2+^ ions, during the repeated chemical charging/galvanostatic discharging cycles^25,34^.

**Fig. 5:**
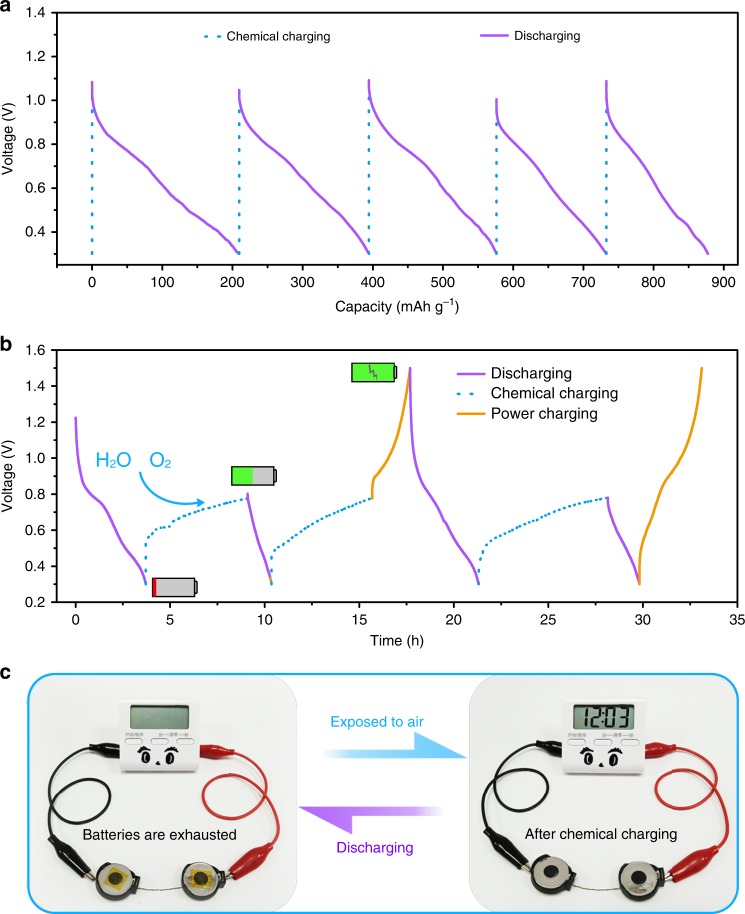
In situ chemical charging behavior of the “open” ZIBs at different modes. **a** Repeated chemical charging/galvanostatic discharging cycles of the ZIBs. **b** Charging/discharging behavior of the ZIBs at chemical or/and galvanostatic charging hybrid modes. **c** A timer powered by two self-charging ZIBs in series at (left) exhausted and (right) chemically charged states.

According to the redox reaction mechanism, O_2_ and H_2_O are essential in the chemical charging process. Considering that water-based electrolyte was utilized in aqueous ZIBs and the Zn anode was stable in ambient air^58–60^, we designed “open” coin-type aqueous ZIBs, where the cathode caps were predrilled with holes to import O_2_ into electrolyte and then reacted with CaZn_3.6_VO to realize the chemical charging process in situ. In such chemically self-charging ZIBs, the CaVO cathode simultaneously serves as an electrode for energy conversion and storage. After the batteries were fully discharged, the gas diffusion window was opened to ensure that O_2_ could continuously diffuse into electrolyte and react with CaZn_3.6_VO cathode. These “open” coin-type ZIBs still exhibit superior self-rechargeability, as reflected by the repeated chemical charging/galvanostatic discharging cycles (Fig. 5a). Furthermore, the chemical charging/galvanostatic discharging process is also reversible. In addition, such chemically self-charging ZIBs can work at multiple charge/discharge modes. When they are chemically charged, the OCV slowly reaches ~0.77 V (Fig. 5b, Supplementary Fig. 28). After the batteries are exhausted, they can be chemically recharged again. Impressively, when the external power supply is available, the batteries can be galvanostatically recharged from self-charged state (0.77 V) to the fully charged state (1.5 V). Furthermore, it is worth mentioning that the operation of chemical charging process has almost no effect on the subsequent galvanostatic charging/discharging process. Similar GCD behavior is observed after several chemical charging cycles (Fig. 5b, Supplementary Fig. 29), indicating the excellent reusability of these chemically self-charging ZIBs. To demonstrate the feasibility of the resultant chemically self-charging ZIBs via a simple visual cue, two “open” coin-type batteries were connected in series to power a timer. After the batteries were exhausted and then exposed to the air, they could be chemically recharged to light up the liquid crystal display from the timer (Fig. 5c), suggesting their availability as chemically self-charging energy storage devices.

## Discussion

The recharging and reuse of commercial batteries is often limited in the harsh environment or remote area, where electrical grid is unavailable. Therefore, self-charging power systems that integrate energy harvesting devices and batteries together must be considered. The rational design of self-charging power systems mainly depends on the fabrication of innovative electrodes and the construction of simplified device configurations. In our case, the favorable nanoribbon morphology, open framework and expanded interlayer spacing endow CaVO with the fast kinetics of Zn^2+^ ion insertion/extraction in the layered structure. As a result, Zn/CaVO batteries exhibit a high initial discharge capacity (300 mAh g^−1^ at 0.1 A g^−1^), excellent rate performance (62 mAh g^−1^ at 30 A g^−1^) and outstanding cycling stability (100% capacity retention after 10,000 cycles). Owing to the redox potential difference between the fully discharged product CaZn_3.6_VO and O_2_, the redox reaction between them will occur, where CaZn_3.6_VO releases electrons to be oxidized and O_2_ accepts these electrons to be reduced simultaneously. Meanwhile, the Zn^2+^ ions are extracted from CaZn_3.6_VO to balance the charge. As a result, the oxidation of vanadium and extraction of the Zn^2+^ ions take place in CaZn_3.6_VO during above redox process at ambient condition. Clearly, the fully discharged product CaZn_3.6_VO recovers to its charged states in such redox process instead of external power. Therefore, Zn/CaZn_3.6−x_VO system can harvest chemical energy from the ambient environment and convert it to electrical energy stored in batteries through the chemical charging process. They can be self-charged to ~1.05 V without any external power supply and deliver a considerable discharge capacity of ~239 mAh g^−1^. Furthermore, the chemical charging/galvanostatic discharging process is also reversible in such open battery design. More importantly, chemically self-charging ZIBs are compatible with different chemical or/and galvanostatic charging hybrid modes. Chemically self-charging ability ensures that Zn/CaVO batteries are available at various conditions. It is different from the conventional ZIBs and zinc–air batteries, which are only charged by external power supply. In the case of rechargeable zinc–air battery^61^, O_2_ is an oxidant and reduced to OH^−^ ions via a reduction reaction at the air electrode during the discharge process. The generated OH^−^ ions then migrate to the zinc electrode, forming insoluble zinc oxide (ZnO) at supersaturated Zn(OH)_2- 4_ concentrations. In its charge process, the OH^−^ ions are oxidized to generate O_2_, which is driven by external power supply. However, in our case, during the discharge process, Zn^2+^ ions insert into the CaVO, which is a characteristic behavior. In this process, O_2_ does not participate in the reaction. During the self-charging process, the discharge product CaZn_3.6_VO will be oxidized by O_2_ due to the difference in redox potential between O_2_ and CaZn_3.6_VO, replacing the electric energy to make the oxidization of discharged product and realizing the self-charging. However, during the chemical charging process, the Zn^2+^ ions will not be redeposited on the Zn anode and the formation of byproduct Zn_x+y_(CF_3_SO_3_)_2y_(OH)_2x_ will consume Zn^2+^ ions from the anode irreversibly, limiting the long-term chemical charging/galvanostatic discharging cycling. Significantly, the Zn_x+y_(CF_3_SO_3_)_2y_(OH)_2x_ can be decomposed by a electrochemical charging process and Zn^2+^ ions could be also redeposited on the Zn anode. This work paves the way for the potential application of ZIBs in self-charging systems, and the chemical charging strategy provides a promising research direction for the self-powered systems.

## Methods

### Materials

Ammonium metavanadate (NH_4_VO_3_, 99%) and N-methyl-2-pyrrolidone (NMP, 99.5%) were purchased from Aladdin. Sodium dodecyl sulfate (SDS, 99%), and polyvinylidene fluoride (PVDF) were purchased from Sinopharm Chemical Reagent Co., Ltd. Single-walled carbon nanotubes (SWCNTs, P3) were purchased from Carbon Solutions Inc. Zn foil (99.9%) was purchased from Alfa Aesar. Glass fiber (Grade GF/A) was from Whatman. Zn (CF_3_SO_3_)_2_, 97.5% was from J&K Scientific Ltd. The prepared electrolyte was purified before using.

### Preparation of CaVO nanoribbons

CaVO nanoribbons were fabricated by a simple hydrothermal method. A total of 117 mg of NH_4_VO_3_ was added into 30 mL deionized water and stirred at 80 °C for 20 min to obtain a clear yellowish solution. Meanwhile, 666 mg of CaCl_2_ and 100 mg SDS were dissolved in another 30 mL deionized water, and stirred at 60 °C for 10 min to achieve a transparent solution. Subsequently, the CaCl_2_/SDS solution was added into NH_4_VO_3_ solution under stirring. Finally, the above mixture solution was transferred into a 100 mL Teflon-lined autoclave and heated at 160 °C for 10 h. After cooling to room temperature, the brownish-red precipitates were washed with deionized water and freeze-dried.

### Electrochemical measurement

Electrochemical performance of CaVO nanoribbons was measured in CR2032 coin cells. The cathode was prepared by mixing the CaVO nanoribbons, SWCNTs, and PVDF in a weight ratio of 7:2:1 by NMP. Then, the slurry was coated on stainless-steel meshes and dried at 60 °C for 12 h under vacuum. The mass loading of CaVO in the cathode is ~1 mg cm^−2^. Commercial glass fiber and zinc foil with a thickness of 30 μm were employed as the separator and anode, respectively. Aqueous 4 M Zn (CF_3_SO_3_)_2_ solution was used as electrolyte since the high-concentration electrolyte would not only alter the solvation and transporting behaviors of cations/anions to enhance stability and kinetics, but also reduce the water activity and water-induced side reactions^48,62–64^. They were assembled into CR2032 coin cells by a traditional method. The GCD tests were performed on a battery test system (LAND CT2001A) with a voltage window of 0.3–1.5 V. CV were measured using an electrochemical workstation (CHI660E) in a voltage window from 0.3 to 1.5 V.

To understand the mechanism of redox reaction between CaZn_3.6_VO and O_2_, the Zn/CaVO batteries were discharged to 0.3 V at a current density of 0.1 A g^−1^. Subsequently, the fully discharged cathodes (CaZn_3.6_VO) were washed by deionized water and then immersed in 4 M Zn(CF_3_SO_3_)_2_ solution or water for different times to react with the dissolved oxygen. After that, the treated CaZn_3.6-x_VO cathodes were assembled into coin cells again for the electrochemical performance measurement. In the in situ chemical charging process, the cathode cap was predrilled with a hole to import oxygen. The cell was first sealed with a Kapton membrane, which was subsequently wiped off to enable O_2_ to pass through the hole. Furthermore, to avoid the excessive volatilization of water from the electrolyte, the chemical charging process was carried out under a high humidity condition (>60%). After self-charging, the cell was sealed again. The specific capacities were calculated based on the mass of the active material CaVO of cathodes.

### Characterization

The morphologies of CaVO nanoribbons and cathodes were characterized via field-emission scanning electron microscopy (SEM, JEOL JSM7500F, 5 kV and Phenom XL, 15 kV). The SEM–energy dispersive spectroscopy (EDS) of the samples are collected with Phenom XL at an acceleration voltage of 15 kV. The microstructure was characterized by TEM (FEI Talos F200X and FEI Talos F200X G2) equipped with EDS mapping at an acceleration voltage of 200 kV. The crystalline structure of samples was determined by XRD (Rigaku SmartLab) with Cu Kα radiation (*λ* = 0.15405 nm). TGA (Netzsch STA 449 F3 Jupiter analyzer) was carried out in an Ar flow from room temperature to 600 °C at a heating rate of 10 °C min^−1^. In situ XRD experiments were performed using home-made cells that were designed with Be window for X-ray penetration. XPS (PerkinElmer PHI 1600 ESCA) was used to characterize the composition and surface oxidation state of the electrodes. V K-edge XANES spectra were collected at the beamline 14W1 in Shanghai Synchrotron Radiation Facility. The solid state ^1^H nuclear magnetic resonance (^1^H NMR) was taken from a 400 MHz superconducting NMR spectrometer (AVANCE ||| 400).

## Supplementary information

Supplementary Information

## Data Availability

The authors declare that all the relevant data are available within the paper and its Supplementary Information file or from the corresponding author upon reasonable request.
