# Supplementary material for: A chemically self-charging aqueous zinc-ion battery
Source: Nat Commun. 2020 May 4;11:2199. doi: 10.1038/s41467-020-16039-5 (PMC7198488; doi:10.1038/s41467-020-16039-5)
Supplement: Supplementary file 1 — Supplementary Information [file 41467_2020_16039_MOESM1_ESM.pdf]

Supplementary Information for

A Chemically Self-Charging Aqueous Zinc-Ion Battery

Zhang et al.

Supplementary Figure

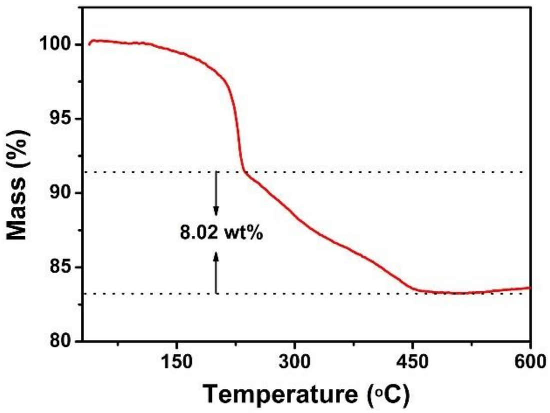

Supplementary Fig. 1 TGA curve of the CaVO sample. Additional discussion is described in Supplementary Note 1.

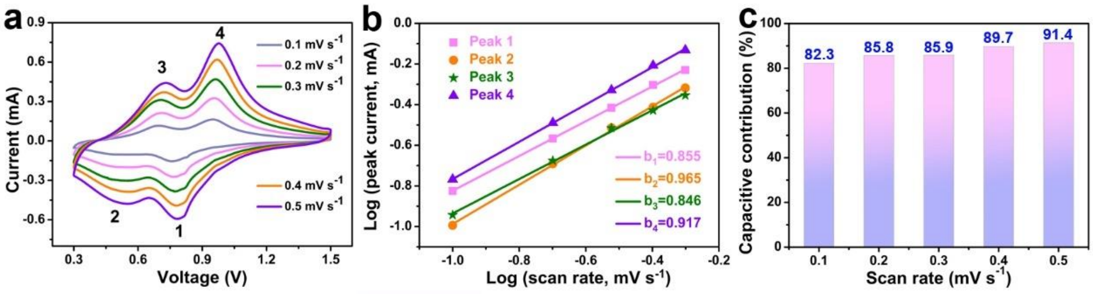

Supplementary Fig. 2 Kinetics analysis of Zn^{2+} ion insertion in CaVO. (**a**) CV curves of the Zn/CaVO batteries at different scan rates and (**b**) the corresponding plots of log (peak current) versus log (scan rate) at different redox peaks. (**c**) The capacitive contributions at different scan rates. Detailed calculation is described in Supplementary Note 2.

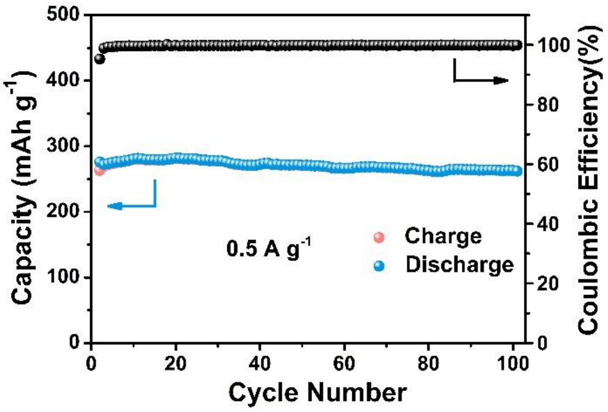

Supplementary Fig. 3 Cycling performance of Zn/CaVO batteries at 0.5 A g^{-1}.

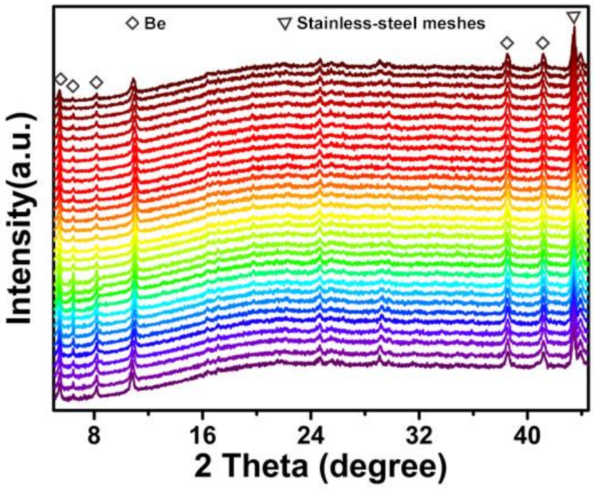

Supplementary Fig. 4 *In-situ* XRD full patterns of Zn/CaVO batteries at 0.2 A g^{-1} during the first cycle.

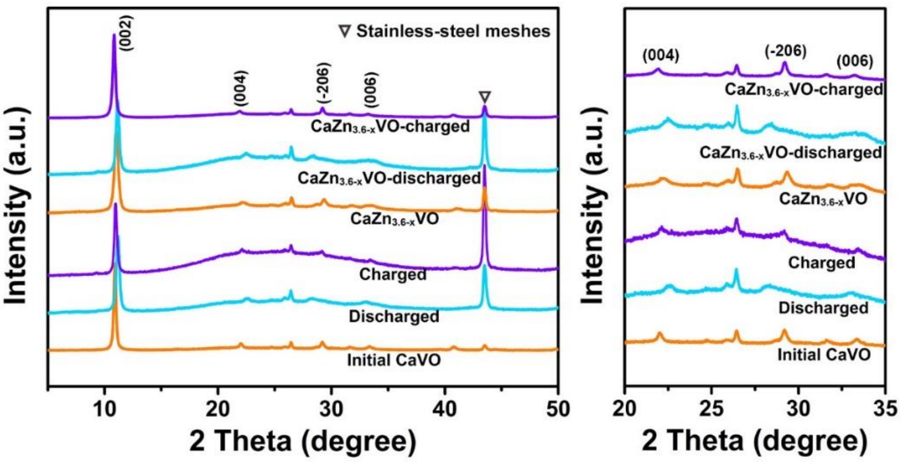

Supplementary Fig. 5 The *ex-situ* XRD patterns of the CaVO-based electrodes at different states in the Zn/CaVO and Zn/$CaZn_{3.6-x}VO$ batteries after the $CaZn_{3.6}VO$ electrode is oxidized by O_2 in 4 M $Zn(CF_3SO_3)_2$ solution for 36 h.

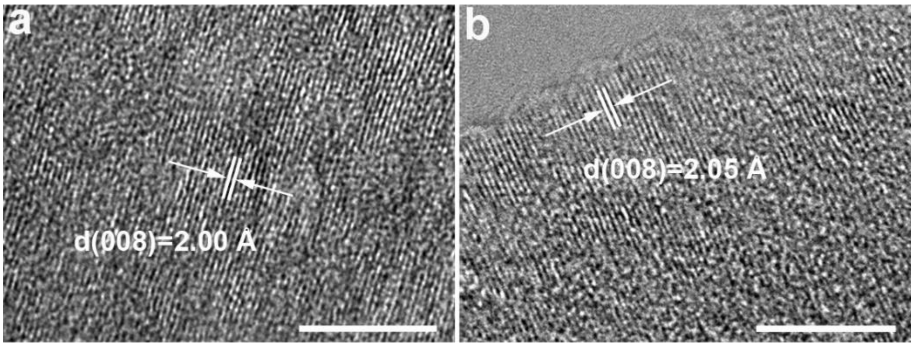

Supplementary Fig. 6 The HRTEM images of the CaVO at different states. The HRTEM images of the CaVO at (**a**) fully discharged and (**b**) fully charged states. Scale bars, 5 nm.

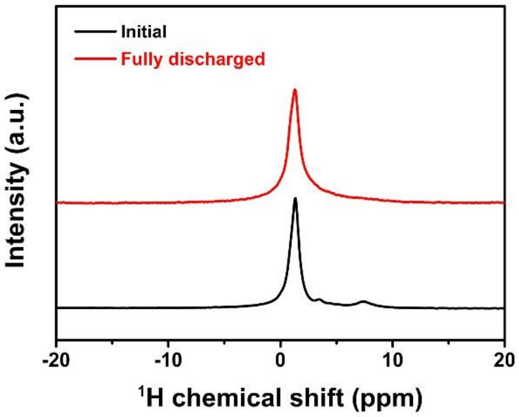

Supplementary Fig. 7 The solid state ^{1}H NMR of the CaVO-based electrode at initial and fully discharged states. Corresponding discussion on whether H^{+} ions participate in ion-insertion reaction in this system is described in Supplementary Note 3.

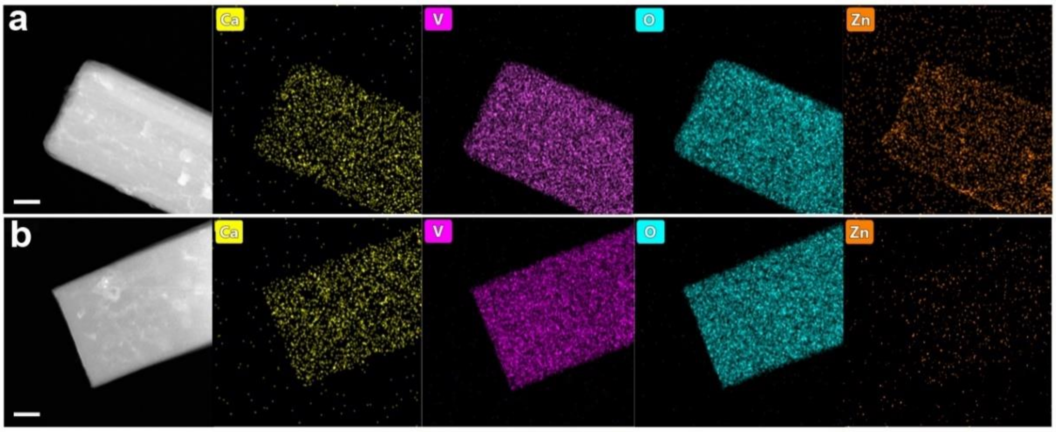

Supplementary Fig. 8 TEM elemental mapping images of CaVO electrodes at different states. TEM elemental mapping images of CaVO electrodes at (**a**) fully discharged and (**b**) fully charged states. Scale bars, 100 nm.

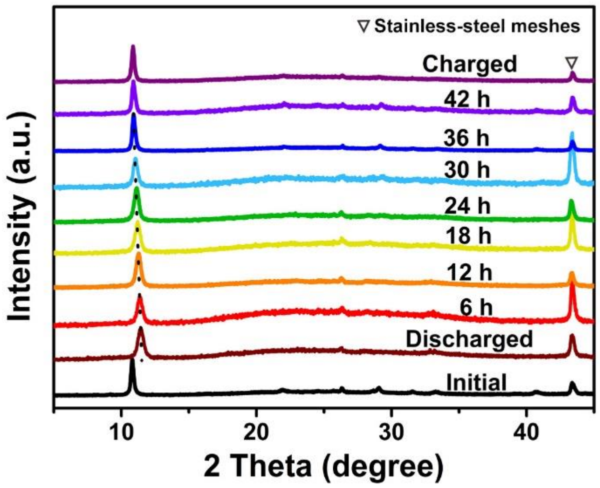

Supplementary Fig. 9 The full XRD patterns of the CaVO electrodes at different states and the $CaZn_{3.6}VO$ electrodes after being oxidized by O_2 in 4 M $Zn(CF_3SO_3)_2$ solution for different times.

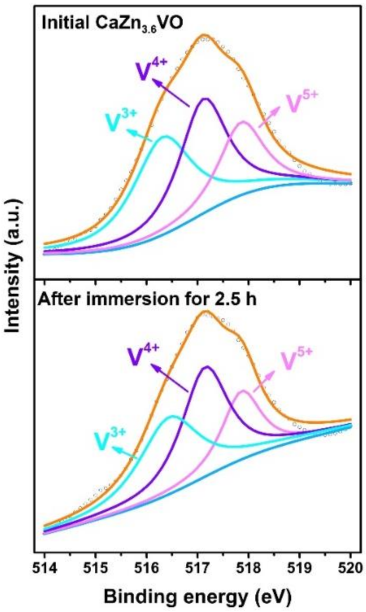

Supplementary Fig. 10 V 2p XPS spectra of the $CaZn_{3.6}VO$ electrode after being immersed in acetonitrile for 2.5 h.

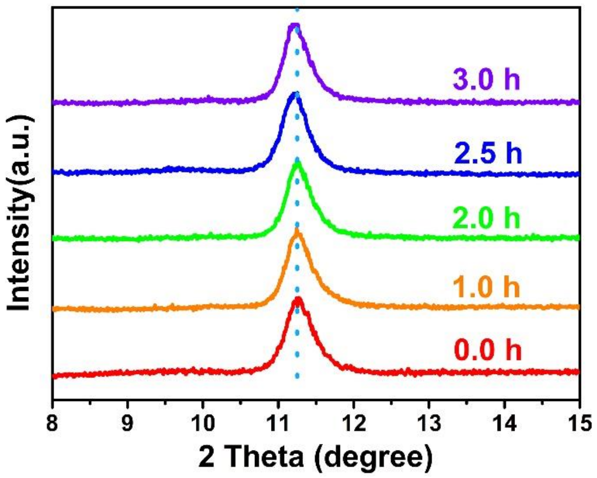

Supplementary Fig. 11 XRD patterns of the $CaZn_{3.6}VO$ electrodes after being immersed in acetonitrile for different times.

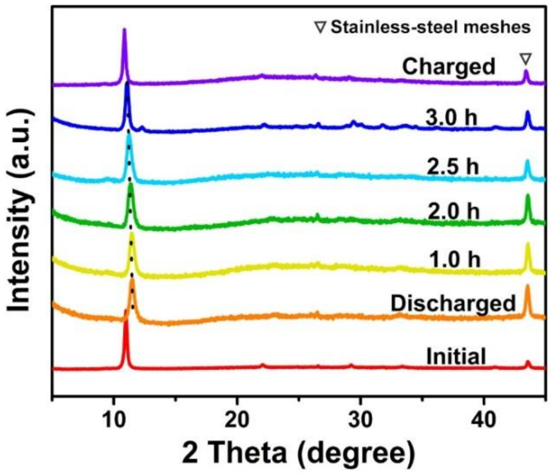

Supplementary Fig. 12 The XRD patterns of the CaVO electrodes at different states and the $CaZn_{3.6}VO$ electrodes after being oxidized by O_2 in deionized water for different times. Detailed discussion on the redox reaction process between $CaZn_{3.6}VO$ and O_2 in the neutral deionized water is described in Supplementary Note 5.

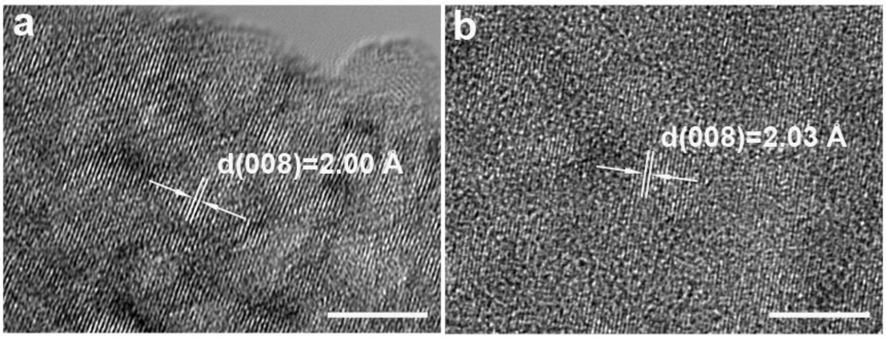

Supplementary Fig. 13 The HRTEM images of the $CaZn_{3.6}VO$ at different states. The HRTEM images of the $CaZn_{3.6}VO$ (**a**) at initial state and (**b**) after oxidation in deionized water for 2.5 h. Scale bars, 5 nm.

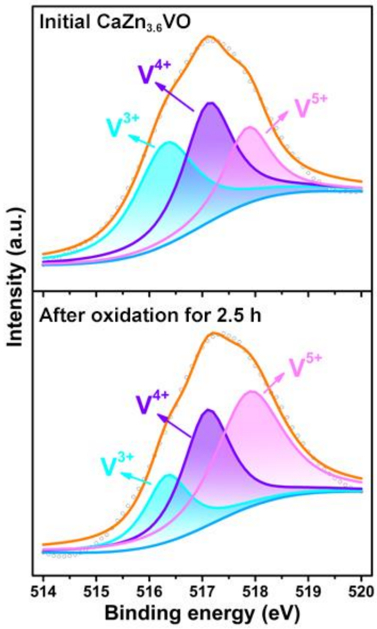

Supplementary Fig. 14 Comparison of XPS spectra of V 2p before and after oxidation in deionized water.

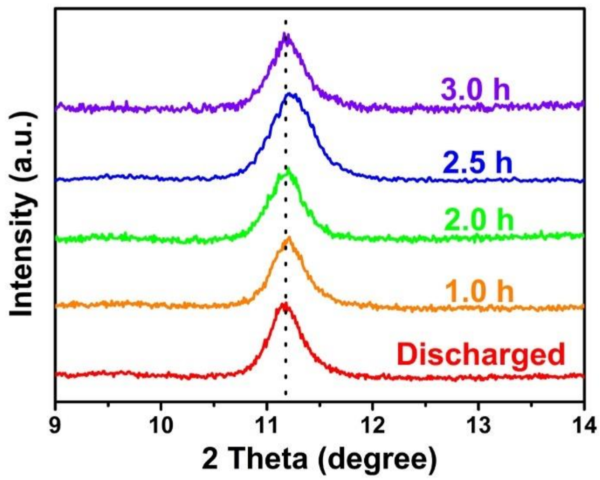

Supplementary Fig. 15 XRD patterns of the $CaZn_{3.6}VO$ electrodes after being immersed in nitrogen-saturated water for different times. Corresponding discussion on ruling out the possibility of the expansion of the interlayer spacing resulting from the intake of water molecules is described in Supplementary Note 6.

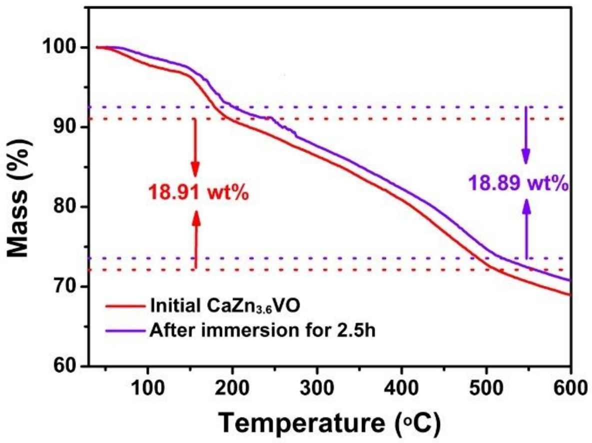

Supplementary Fig. 16 The TGA curves of the $CaZn_{3.6}VO$ electrodes at initial state and after immersion in water with dissolved oxygen for 2.5 h. Corresponding discussion is described in Supplementary Note 6.

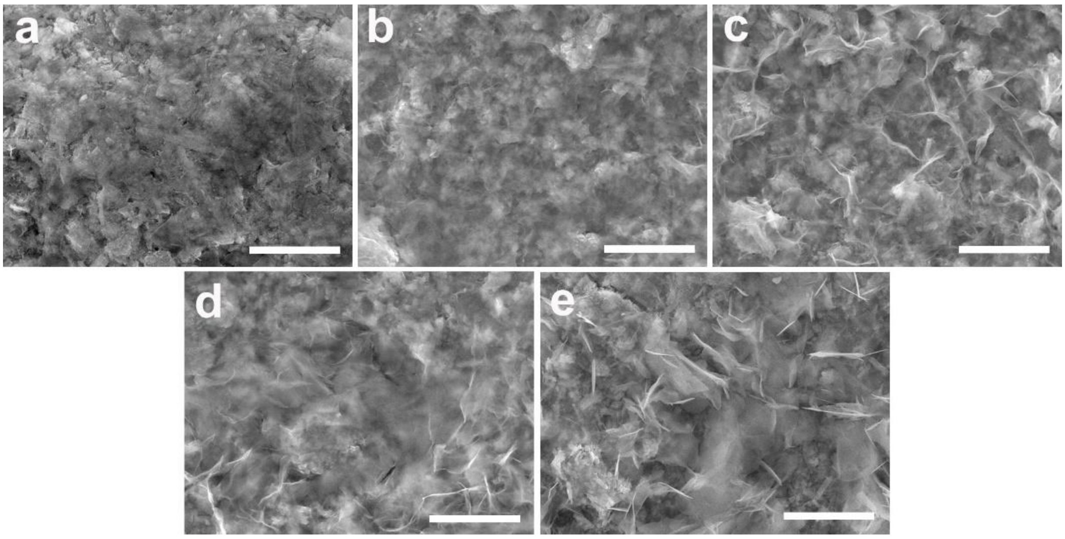

Supplementary Fig. 17 Morphology characterization of $CaZn_{3.6}VO$ electrodes at different states. SEM images of $CaZn_{3.6}VO$ electrodes after oxidation in deionized water for different times: (**a**) 0 h, (**b**) 1 h, (**c**) 2 h, (**d**) 2.5 h, (**e**) 3 h. Scale bars, 10 μm.

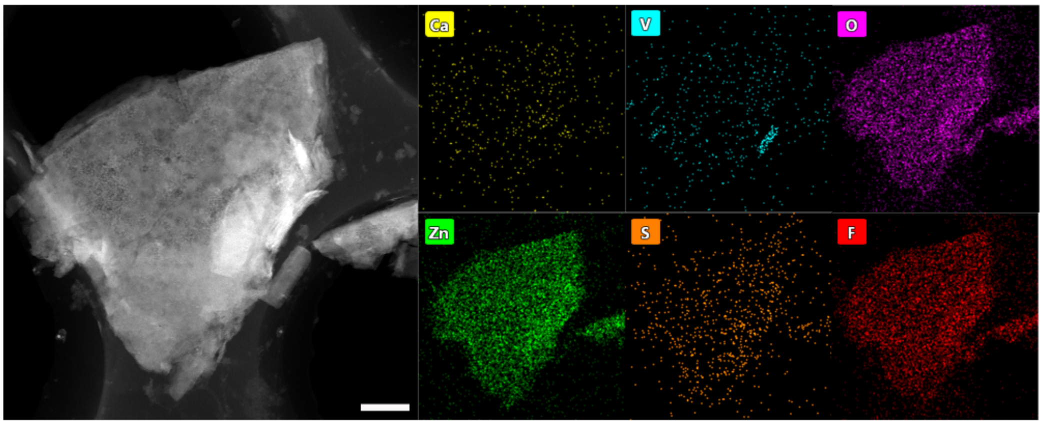

Supplementary Fig. 18 TEM elemental mapping images of $Zn_{x+y}(CF_3SO_3)_{2y}(OH)_{2x}$ flake after the $CaZn_{3.6}VO$ electrode is oxidized by O_2 in deionized water for 2.5 h. Only Zn, S, F, and O are detected, but V is not observed, indicating the existence of $Zn_{x+y}(CF_3SO_3)_{2y}(OH)_{2x}$. Scale bars, 200 nm.

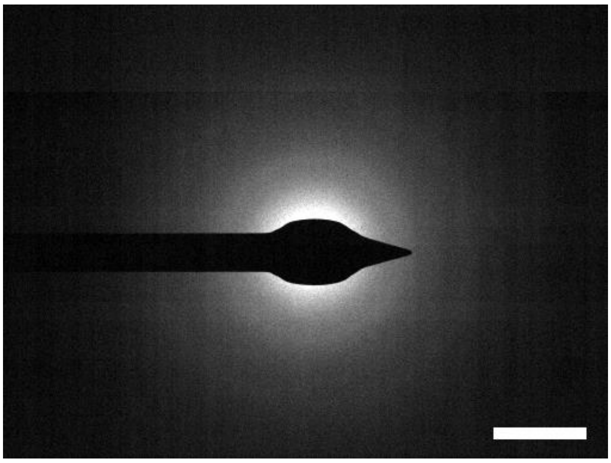

Supplementary Fig. 19 The selected area electron diffraction (SAED) pattern of $Zn_{x+y}(CF_3SO_3)_{2y}(OH)_{2x}$. Scale bars, 5 1/nm. Corresponding discussion is described in Supplementary Note 7.

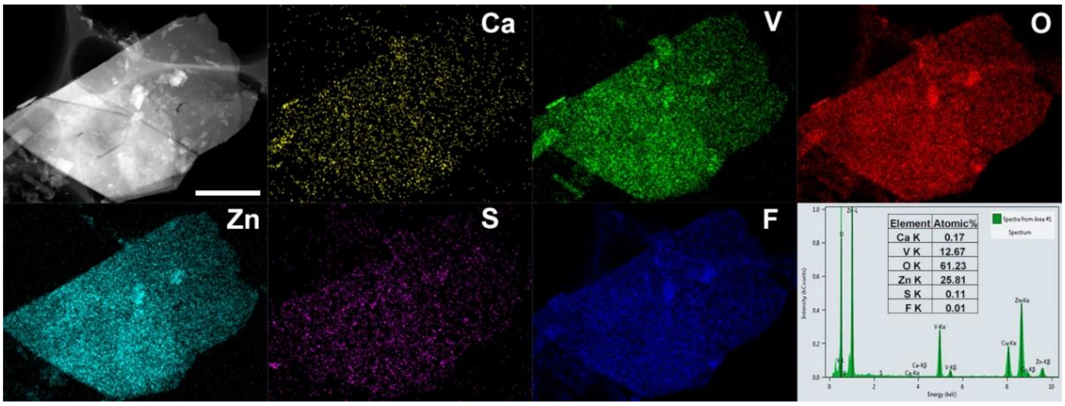

Supplementary Fig. 20 TEM elemental mapping images and EDS analysis of $Zn_3V_2O_7(OH)_2 \cdot 2H_2O$ flake that was obtained by immersing the $CaZn_{3.6}VO$ electrode in water for 3 h. The flake consists of Zn, V and O without S and F, further confirming the formation of $Zn_3V_2O_7(OH)_2 \cdot 2H_2O$. Scale bars, 1 μm. Corresponding explanation about the formation of $Zn_3V_2O_7(OH)_2 \cdot 2H_2O$ is described in Supplementary Note 8.

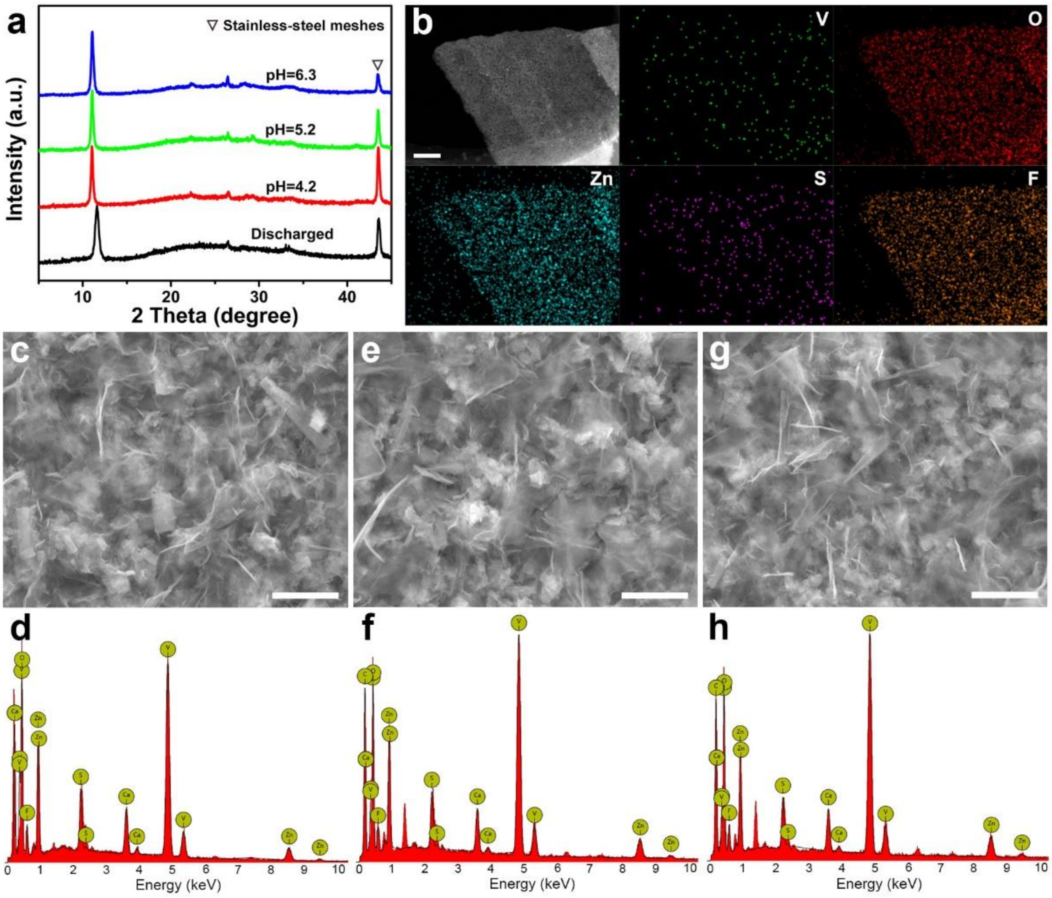

Supplementary Fig. 21 Characterization of $CaZn_{3.6}VO$ electrodes after oxidation in the water with different pH for 2 h. (**a**) The XRD patterns of resultant $CaZn_{3.6}VO$ electrodes. (**b**) TEM elemental mapping images of $Zn_{x+y}(CF_3SO_3)_{2y}(OH)_{2x}$ flakes after the $CaZn_{3.6}VO$ electrode is oxidized for 2 h in the water with pH value of 4.2. The SEM images and EDS patterns of $CaZn_{3.6}VO$ electrodes after oxidation in the water with different pH for 2 h: (**c**, **d**) pH = 4.2, (**e**, **f**) pH = 5.2, (**g**, **h**) pH = 6.3. Scale bars: (**b**) 200 nm; (**c**, **e**, **g**) 4 μm. Corresponding discussion on the dependence of the $Zn_{x+y}(CF_3SO_3)_{2y}(OH)_{2x}$ formation on the pH is described in Supplementary Note 9.

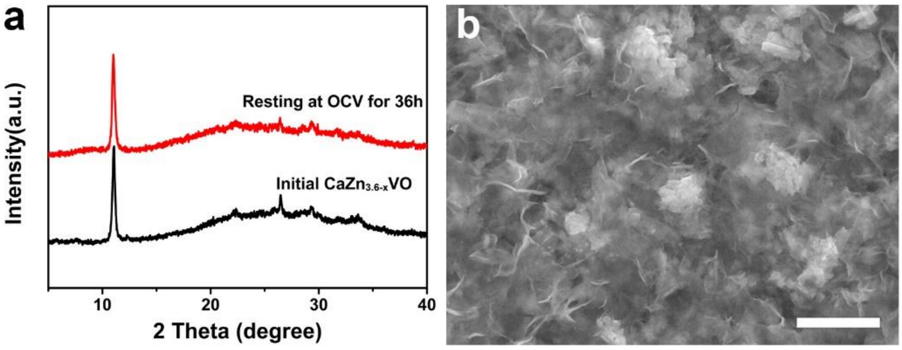

Supplementary Fig. 22 Characterization of chemically charged $CaZn_{3.6-x}VO$ electrodes after resting at OCV. (**a**) Comparison of XRD patterns of $CaZn_{3.6-x}VO$ electrodes (oxidation in deionized water for 2.5 h) before and after resting at OCV for 36 h. (**b**) SEM image of $CaZn_{3.6-x}VO$ electrode (oxidation in deionized water for 2.5 h) after resting at OCV for 36 h. Scale bars, 4 μm. Corresponding discussion is described in Supplementary Note 10.

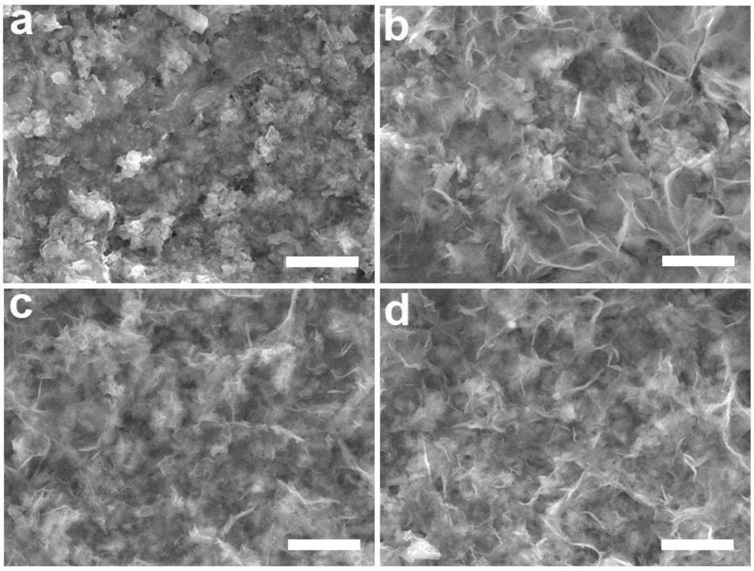

Supplementary Fig. 23 Morphology characterization of $CaZn_{3.6}VO$ electrodes at different states. SEM images of $CaZn_{3.6}VO$ electrodes after oxidation in 4 M $Zn(CF_3SO_3)_2$ solution for different times: (**a**) 0 h, (**b**) 18 h, (**c**) 36 h, (**d**) 42 h. Scale bars, 5 μm.

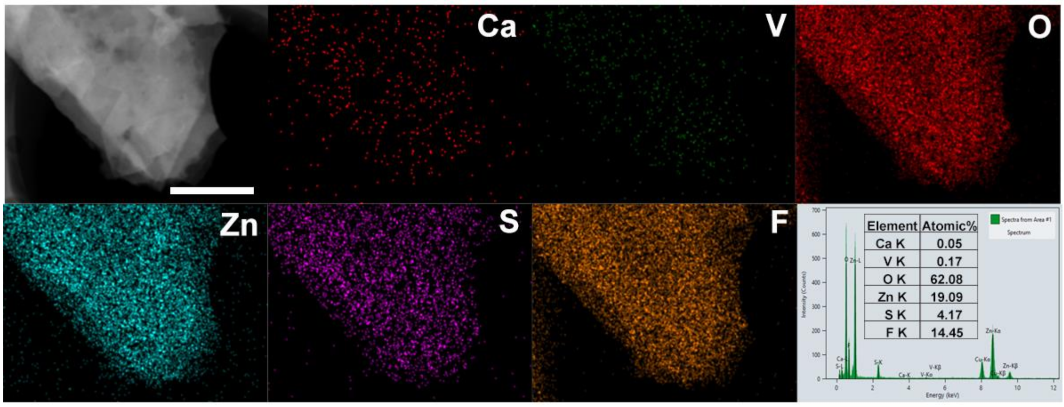

Supplementary Fig. 24 TEM elemental mapping images and EDS analysis of $Zn_{x+y}(CF_3SO_3)_{2y}(OH)_{2x}$ flakes after the $CaZn_{3.6}VO$ electrode is oxidized by O_2 in 4 M $Zn(CF_3SO_3)_2$ solution for 36 h. The flake consists of Zn, S, F and O without V, indicating that flake is ascribed to the $Zn_{x+y}(CF_3SO_3)_{2y}(OH)_{2x}$. Scale bars, 1 μm.

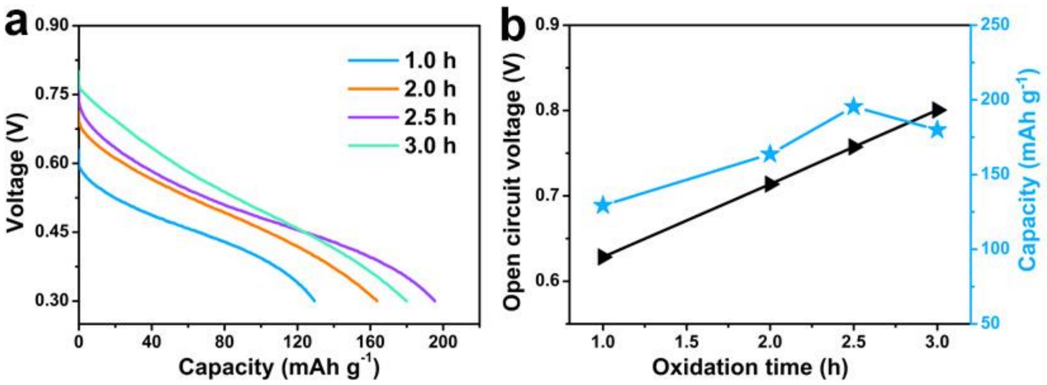

Supplementary Fig. 25 Electrochemical performance of Zn/$CaZn_{3.6-x}$VO batteries after the $CaZn_{3.6}$VO electrodes are oxidized in deionized water for different times. (**a**) The galvanostatic discharge curves of Zn/$CaZn_{3.6-x}$VO batteries at 0.1 A g^{-1}. (**b**) Effect of the oxidation time on OCV and discharge capacity of Zn/$CaZn_{3.6-x}$VO batteries. Corresponding discussion is described in Supplementary Note 11.

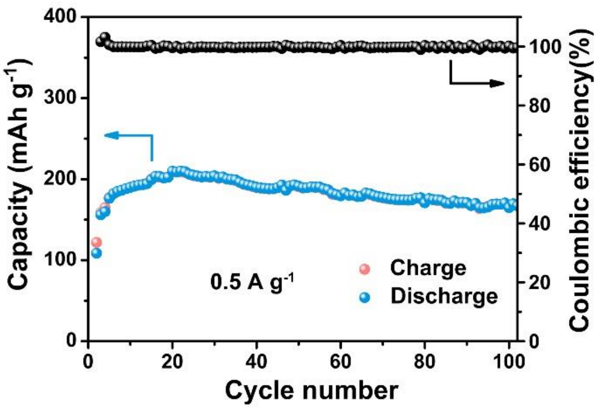

Supplementary Fig. 26 Cycling performance of Zn/$CaZn_{3.6-x}VO$ battery at 0.5 A g^{-1} after the $CaZn_{3.6}VO$ electrode is oxidized in deionized water for 3 h. Corresponding discussion is described in Supplementary Note 11.

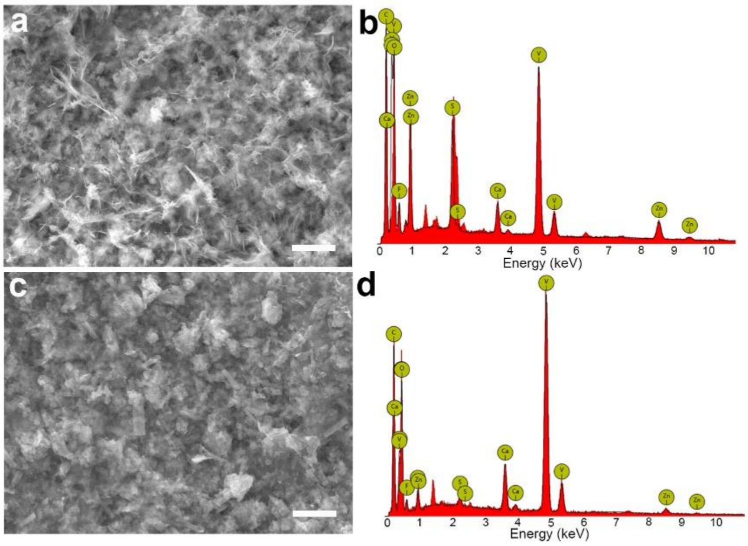

Supplementary Fig. 27 Morphology and chemical composition characterization of $CaZn_{3.6-x}VO$ electrodes. The SEM images and corresponding EDS patterns of the chemically charged $CaZn_{3.6-x}VO$ electrodes (**a**, **b**) before and (**c**, **d**) after electrochemical charging. Scale bars, 5 μm. Corresponding discussion is described in Supplementary Note 12.

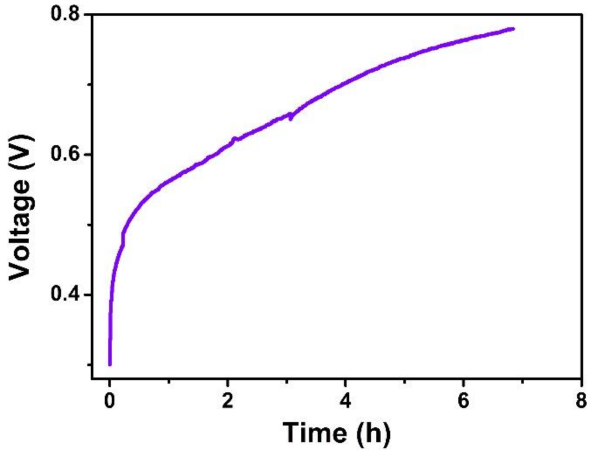

Supplementary Fig. 28 Voltage-time curves of open coin-type Zn/$CaZn_{3.6-x}VO$ batteries during the *in-situ* chemical charging process.

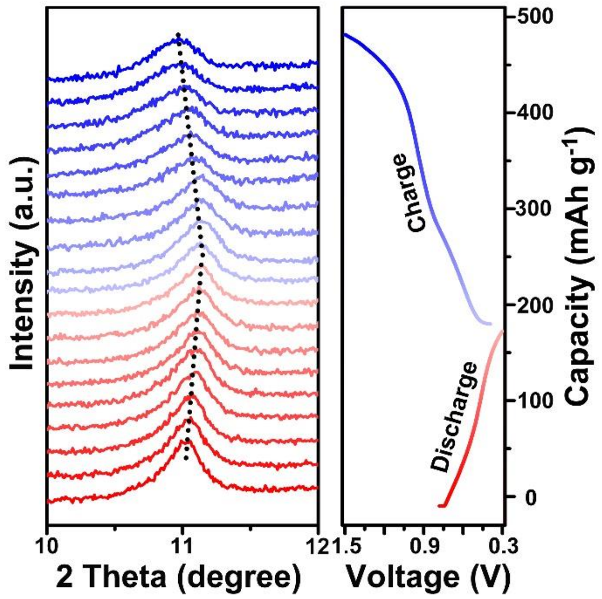

Supplementary Fig. 29 *In-situ* XRD patterns of (002) reflection of the Zn/$CaZn_{3.6-x}VO$ battery after being chemically charged to 0.77 V and corresponding GCD curves at 0.1 A g^{-1}.

Supplementary Notes

Supplementary Note 1. TGA of the CaVO sample

As shown in Supplementary Fig. 1, TGA displays that there is a weight loss of 8.02 wt% in the temperature range of 230-450 ℃. It suggests that 3 molecular waters exist in a formula unit, which agrees with the theoretical content in CaVO (8.23 wt%).

Supplementary Note 2. Kinetics analysis of Zn^{2+} ion insertion in CaVO

The power-law relationship between peak current (*i*) and scan rate (*v*) can be described as below

$$i = av^b \tag{1}$$

where *a* and *b* are adjustable parameters. By plotting log(*i*) as a function of log(*v*), the *b*-values calculated from the curves approximate to 1 (Supplementary Fig. 2b), indicating that the kinetics of Zn^{2+} ions in CaVO are surface-controlled. Moreover, at a certain scan rate, the surface-controlled contribution (k_1v) and diffusion-controlled contribution ($k_2v^{1/2}$) can be described as below

$$i=k_1v+k_2v^{1/2} \tag{2}$$

Consequently, the results show that the surface-controlled contribution ratio increases from 82.3% to 91.4% with the scan rate rising from 0.1 to 0.5 mV s^{-1} (Supplementary Fig. 2c), reflecting that the surface-controlled contribution holds dominant position.

Supplementary Note 3. Discussion on whether H^+ ions participate in ion-insertion reaction in this system

In the layered CaVO, interlayer water molecules not only bond to Ca atoms, but also interact with the V_6O_{16} layers through hydrogen bonding[1, 2]. Therefore, hydrogen in the CaVO exists in different chemical environments, corresponding to the several resonances in 0-10 ppm region in the ^1H spectrum of the as-prepared CaVO (Supplementary Fig. 7). After the discharge process, the Zn^{2+} ions insert into the interlayer of CaVO, which will influence the chemical environment of hydrogen. As a result, the ^1H spectrum at discharged state is changed in comparison with the case at initial state. No new peak is observed in the ^1H spectrum of the fully discharged product except a very small right-hand tail. In addition, in general, the intercalation of H^+ ions will lead to the formation of $Zn_{x+y}(CF_3SO_3)_{2y}(OH)_{2x}$ precipitate on the discharged electrode in $Zn(CF_3SO_3)_2$ aqueous electrolyte, as suggested by previously reported work[3, 4]. However, in our case, no obvious $Zn_{x+y}(CF_3SO_3)_{2y}(OH)_{2x}$ flake is observed on the discharged electrode, as shown in SEM images (Supplementary Fig. 17a and 23a). Furthermore, no XRD diffraction peaks of $Zn_{x+y}(CF_3SO_3)_{2y}(OH)_{2x}$ located at 6.6°, 13.2°, 19.8°, 26.3° and 33.2° are found in the XRD pattern of discharged electrode (Supplementary Fig. 5). Therefore, these could suggest that H^+ ions would not participate in ion-insertion reaction in this system.

Supplementary Note 4. The analysis of XRD pattern of the $CaZn_{3.6}VO$ electrode after oxidation in 4 M $Zn(CF_3SO_3)_2$ solution for 36 h

During the chemical charging process, the $CaZn_{3.6}VO$ is oxidized by O_2 and Zn^{2+} ions are extracted from the layered structure simultaneously, resulting in the increase of the interlayer spacing of (002) and (004) plane. Therefore, the (002) and (004) reflections shift toward low degree in the corresponding XRD patterns (Supplementary Fig. 5). In addition, the (-206) reflection shifts toward high degree due to the oxidation of V^{4+}/ V^{3+} upon Zn^{2+} ion extraction and the resultant decrease in the V-V bond distance between the layers. However, in this chemically charged state, the broadening of (-206) and (006) reflection is still observed since the structure of $CaZn_{3.6-x}VO$ can not return to its original state completely.

Supplementary Note 5. The redox reaction process between $CaZn_{3.6}VO$ and O_2 in the neutral deionized water

During the redox reaction process in the neutral deionized water, the intercalated Zn^{2+} ions are extracted from the layered structure to balance the charge of $CaZn_{3.6}VO$ since the vanadium in $CaZn_{3.6}VO$ is oxidized, resulting in the increase of the interlayer spacing of $CaZn_{3.6}VO$, as proved by the XRD pattern (Supplementary Fig. 12). When the oxidation time exceeds 2.5 h, several new peaks located at 12.2°, 30.0°, 31.8° and 36.1° can be observed, which match well with the byproduct $Zn_3V_2O_7(OH)_2 \cdot 2H_2O$. Since the irreversible $Zn_3V_2O_7(OH)_2 \cdot 2H_2O$ would result in the battery degradation (see detailed analysis in Supplementary Note 11), the oxidation time in deionized water should be no more than 2.5 h. Meanwhile, the interlayer spacing expansion of the $CaZn_{3.6}VO$ after oxidation is also observed by the HRTEM images (Supplementary Fig. 13). The extraction of Zn^{2+} ions is driven by the valence change of vanadium in $CaZn_{3.6}VO$, as suggested by the XPS spectra (Supplementary Fig. 14), where the intensity of V^{4+} and V^{5+} peaks is significantly enhanced and V^{3+} peak weakens in comparison with those of initial $CaZn_{3.6}VO$.

Supplementary Note 6. Ruling out the possibility of the expansion of the interlayer spacing resulting from the intake of water molecules

In order to rule out the possibility that the expansion of the interlayer spacing could happen due to the intake of water molecules from the electrolyte during charging process, the $CaZn_{3.6}VO$ electrodes were immersed in nitrogen-saturated water, where O_2 was removed. In such case, the redox reaction could not take place due to the absence of O_2. It is noted that the (002) reflection of the resultant $CaZn_{3.6}VO$ electrodes remains unchanged in position (Supplementary Fig. 15), suggesting that the interlayer spacing expansion during charge process is not caused by the water insertion. In addition, the water contents of the discharged and self-charged electrodes were quantified by TGA (Supplementary Fig. 16). The results show that the water contents of the two samples are similar, further confirming that the expansion of the interlayer spacing is ascribed to the insertion/extraction of Zn^{2+} ions rather than the intake of water molecules from the electrolyte.

Supplementary Note 7. The amorphous $Zn_{x+y}(CF_3SO_3)_{2y}(OH)_{2x}$

It can be concluded from Supplementary Fig. 19 that the $Zn_{x+y}(CF_3SO_3)_{2y}(OH)_{2x}$ generated in this chemical charging process is amorphous. It is ascribed to the larger size and anisotropic molecular structure of the triflate anion [3].

Supplementary Note 8. Explanation about the formation of $Zn_3V_2O_7(OH)_2 \cdot 2H_2O$

With the extension of oxidation time, the electrode potential of $CaZn_{3.6-x}VO$ electrode is gradually increased due to the deeper oxidation of vanadium and the extraction of more Zn^{2+} ions. As a result, the redox potential difference (*ΔE*) between $CaZn_{3.6-x}VO$ and O_2 is gradually decreased along with the extension of oxidation time. When the oxidation time exceeds 2.5 h, the reduced *ΔE* will not drive the extraction of Zn^{2+} ions from the $CaZn_{3.6-x}VO$ to form $Zn_{x+y}(CF_3SO_3)_{2y}(OH)_{2x}$. However, since the difference in redox potential between O_2 and $CaZn_{3.6}VO$ still exists, the $CaZn_{3.6-x}VO$ could continue to be oxidized by O_2, and a byproduct $Zn_3V_2O_7(OH)_2 \cdot 2H_2O$ is irreversibly formed (Supplementary Fig. 12 and 20).

Supplementary Note 9. The dependence of the $Zn_{x+y}(CF_3SO_3)_{2y}(OH)_{2x}$ formation on the pH

To understand the dependence of the $Zn_{x+y}(CF_3SO_3)_{2y}(OH)_{2x}$ formation on the pH, the discharged electrodes were immersed into water with different pH values in a mild acidic range (pH= 4.2, 5.2 and 6.3, respectively) for 2 h, which was adjusted by the addition of HCl solution. The pH was maintained at a constant value in this process. After oxidation for 2 h, the (002) reflection in the XRD patterns of resultant $CaZn_{3.6}VO$ gradually shifts toward lower degree (Supplementary Fig. 21a), indicating that the redox reaction between $CaZn_{3.6}VO$ and O_2 can occur in these systems. When the redox reaction takes place in the solution with higher pH, the consumption of H^+ ions results in the faster increase in the local pH at electrode/electrolyte interface, leading to the formation of more $Zn_{x+y}(CF_3SO_3)_{2y}(OH)_{2x}$ (Supplementary Fig. 21c-h). The results indicate that the formation of $Zn_{x+y}(CF_3SO_3)_{2y}(OH)_{2x}$ would depend on the local pH change at the electrode/electrolyte interface during chemical charging process. In addition, it is noted that even at pH of 4.2, $Zn_{x+y}(CF_3SO_3)_{2y}(OH)_{2x}$ can remain flake-like morphology and its chemical composition is similar with the case at pH of 6.3, as suggested by the TEM elemental mapping images and SEM-EDS patterns (Supplementary Fig. 21b, d and h). Therefore, the $Zn_{x+y}(CF_3SO_3)_{2y}(OH)_{2x}$ phase is stable at the pHs from 4.2 to 6.3.

Supplementary Note 10. Characterization of chemically charged $CaZn_{3.6-x}VO$ electrodes after resting at OCV

The chemically charged $CaZn_{3.6-x}VO$ electrode (oxidation for 2.5 h in deionized water) was assembled in a cell and rested at OCV for 36 h. After that, we performed the XRD diffraction of this $CaZn_{3.6-x}VO$ electrode, as shown in Supplementary Fig. 22a. After resting for 36 h, the XRD pattern of $CaZn_{3.6-x}VO$ electrode remains almost unchanged compared with the case before resting. Moreover, after resting for 36 h, the $Zn_{x+y}(CF_3SO_3)_{2y}(OH)_{2x}$ would not further form on or drop from the electrode (Supplementary Fig. 22b).

Supplementary Note 11. Electrochemical performance of Zn/CaZn$_{3.6-x}$VO batteries after the CaZn$_{3.6}$VO electrodes are oxidized in deionized water for different times

As shown in Supplementary Fig. 25, the OCV of the Zn/CaZn$_{3.6-x}$VO batteries rises continually along with extending the oxidation time of CaZn$_{3.6}$VO. When the CaZn$_{3.6}$VO is oxidized for 2.5 and 3 h, the OCV of Zn/CaZn$_{3.6-x}$VO batteries reaches up to 0.76 and 0.80 V, respectively, which is higher than the average platform voltage of Zn/CaVO system. In addition to OCV, the discharge capacity of Zn/CaZn$_{3.6-x}$VO batteries also depends on the oxidation time of CaZn$_{3.6}$VO. When the oxidation time is less than 2.5 h, the discharge capacity gradually increases with the prolongation of oxidation time and a discharge capacity of 195.4 mAh g^{-1} is achieved at 2.5 h. However, it is noted that the discharge capacity of Zn/CaZn$_{3.6-x}$VO batteries would degrade when the oxidation time exceeds 2.5 h as a result of the irreversible formation of $Zn_3V_2O_7(OH)_2 \cdot 2H_2O$ (Supplementary Fig. 12 and 20; Supplementary Note 8). In this system, the flake-like and insulated $Zn_3V_2O_7(OH)_2 \cdot 2H_2O$ derived from the irreversible oxidation of CaZn$_{3.6-x}$VO would be electrochemically inactive[5-7]. Furthermore, the formation of $Zn_3V_2O_7(OH)_2 \cdot 2H_2O$ results in the reduce of redox active material, leading to capacity decay.

Since the $Zn_3V_2O_7(OH)_2 \cdot 2H_2O$ is insulated and displays a flake-like morphology, the formation of $Zn_3V_2O_7(OH)_2 \cdot 2H_2O$ would inevitably increase electrochemical impedance and hinder the insertion of Zn^{2+} ions during subsequent cycles. As a result, the capacity would be degraded in the long-term cycling[6, 7] (Supplementary Fig. 26).

Supplementary Note 12. Morphology and chemical composition characterization of $CaZn_{3.6-x}VO$ electrodes before and after electrochemical charging

After electrochemical charging, the flake-like $Zn_{x+y}(CF_3SO_3)_{2y}(OH)_{2x}$ would be decomposed and disappear from the electrode, as shown in the SEM images (Supplementary Fig. 27a and c). Meanwhile, the signals of Zn and S are significantly reduced in the SEM-EDS pattern of the electrochemically charged electrode compared with the case of chemically charged electrode (Supplementary Fig. 27b and d), which further indicates that $Zn_{x+y}(CF_3SO_3)_{2y}(OH)_{2x}$ is decomposed during the electrochemical charging process.

Supplementary References

1. Kong, L., Shao, M., Xie, Q., Liu, J. & Qian, Y. Hydrothermal growth of single-crystal $CaV_6O_{16}\cdot 3H_2O$ nanoribbons. *J. Cryst. Growth* **260**, 435-439 (2004).
2. Zhang, X. *et al.* Ultralong metahewettite $CaV_6O_{16}\cdot 3H_2O$ nanoribbons as novel host materials for lithium storage: Towards high-rate and excellent long-term cyclability. *Nano Energy* **22**, 38-47 (2016).
3. Oberholzer, P., Tervoort, E., Bouzid, A., Pasquarello, A. & Kundu, D. Oxide versus nonoxide cathode materials for aqueous Zn batteries: An insight into the charge storage mechanism and consequences thereof. *ACS Appl. Mater. Interfaces* **11**, 674-682 (2019).
4. Wang, L., Huang, K.-W., Chen, J. & Zheng, J. Ultralong cycle stability of aqueous zinc-ion batteries with zinc vanadium oxide cathodes. *Sci. Adv.* **5**, eaax4279 (2019).
5. Jo, J. H., Sun, Y.-K. & Myung, S.-T. Hollandite-type Al-doped $VO_{1.52}(OH)_{0.77}$ as a zinc ion insertion host material. *J. Mater. Chem. A* **5**, 8367-8375 (2017).
6. Tang, B., Shan, L., Liang, S. & Zhou, J. Issues and opportunities facing aqueous zinc-ion batteries. *Energy Environ. Sci.* **12**, 3288-3304 (2019).
7. Yang, Y. et al. Transition metal ion-preintercalated V_2O_5 as high-performance aqueous zinc-ion battery cathode with broad temperature adaptability. *Nano Energy* **61**, 617-625 (2019).
